# Phase-specific learning curves in robot-assisted rectal surgery: implications for surgical training

**DOI:** 10.1007/s00384-025-05015-4

**Published:** 2025-10-11

**Authors:** Ryota Ito, Takeru Matsuda, Hiroshi Hasegawa, Yasunori Otowa, Masayuki Ando, Yasufumi Koterazawa, Naoki Urakawa, Hironobu Goto, Shingo Kanaji, Yoshihiro Kakeji

**Affiliations:** 1 Division of Gastrointestinal Surgery, Department of Surgery, Kobe University Graduate School of Medicine, Kobe, Japan; 2 Division of Minimally Invasive Surgery, Department of Surgery, Kobe University Graduate School of Medicine, 7-5-2 Kusunoki-Chou, Chuo-Ku, Kobe, 650-0017 Japan

**Keywords:** Rectal cancer, Robot-assisted surgery, Learning curve, CUSUM

## Abstract

**Purpose:**

Techniques for abdominal and pelvic manipulation vary in robot-assisted rectal surgery (RRS); thus, differences in the learning period between these two phases remain unclear.

**Methods:**

This retrospective single-center study included 75 patients diagnosed with rectal malignancy who underwent RRS from September 2019 to May 2024. The cumulative sum method was used to analyze the learning curve and compare between abdominal and pelvic phases.

**Results:**

The learning curve for the total console time consisted of three phases: I (learning phase, cases 1–25), II (consolidation phase, cases 26–55), and III (maturing phase, cases 56–71). The learning curve for the abdominal phase involved two phases: I (learning phase, cases 1–23) and II (maturing phase, cases 24–71), with no consolidation phase observed. Conversely, in the pelvic phase, the learning phase was achieved in 27 cases, followed by the consolidation phase in 32 cases (cases 28–59) and the maturing phase in the last 16 cases (cases 60–75).

**Conclusions:**

The learning period during RRS was shorter for the abdominal manipulation than for the pelvic manipulation, considering the absence of the consolidation phase. Education and training that consider these points may be useful for the more efficient learning of RRS.

## Introduction

Minimally invasive surgery (MIS) has become a mainstream approach for rectal malignancy in recent years because it results in fewer postoperative complications and blood loss than open surgery [1]. The proportion of robot-assisted rectal surgery (RRS) in MIS has been increasing since the first appearance of telesurgical robots in the late 1990 s [2]. Reportedly, RRS has advantages such as autonomic nerve preservation and less blood loss due to the improved stability of the procedure in the pelvis [3, 4]. Therefore, learning RRS efficiently is crucial not only for the expert but also for the junior surgeons.

Some of the published data suggests that there is a shorter learning curve for RRS than for laparoscopic rectal surgery [5, 6]. RRS remains in the process of being standardized; however, it requires abdominal manipulation, such as the inferior mesenteric artery (IMA) processing, and pelvic manipulation, such as rectal mobilization. In general, the abdominal manipulation included IMA dissection and mobilization of the descending-sigmoid colon, whereas the pelvic manipulation included rectal mobilization and total mesorectal excision (TME) [7]. However, the nature of the abdominal and pelvic manipulation techniques is different. Further, differences in the learning period between the abdominal and pelvic phases remain unclear. In this study, we categorized the console time of RRS into the abdominal and pelvic phases and examined the differences in the acquisition process of each manipulation using the cumulative sum (CUSUM) method.

## Methods

### Patients

This retrospective single-center study involved surgeries performed from September 2019 to May 2024 by three surgeons (TM, HH, and RS) who met the requirements of the Japan Society for Endoscopic Surgery endoscopic surgical skill qualification system. A total of 152 patients with rectal malignancy underwent RRS. This study excluded 60 patients undergoing transanal TME, 13 patients with missing data, 3 patients with atypical robotic procedures (combined resection of the bladder, change of surgical technique due to peritoneal dissemination, and subtotal resection of the colon), and 1 patient undergoing conversion to laparoscopy. Finally, this study included 75 cases. All clinical, pathological, and operative data were collected from medical records. The surgeries were performed with the da Vinci X or Xi Surgical System® (Intuitive Surgical, Sunnyvale, CA) or the hinotori™ Surgical Robot System (Medicaroid Cooperation, Kobe, Japan). The institutional review board (IRB) and Ethics Committee of Kobe University Graduate School of Medicine approved this study (IRB reference code: B240119). This study adhered to the STROBE (Strengthening the Reporting of Observational Studies in Epidemiology) guidelines.

### Surgical procedures

The surgeries were performed based on the procedure previously described by Nozawa et al. [8]. In brief, the operations were initiated with the lithotomy position. The first arm port was positioned in the left upper abdomen, the second in the umbilical region, and the third and fourth in the right lower abdomen so that all were in a straight line. To assist with rectal traction, 1 or 2 ports were placed in the right upper abdomen. The robot was rolled in from the patient’s left side and the targeting was performed for the SD junction. After docking the patient cart, dissection around the IMA using a medial-to-lateral approach and mobilization of the sigmoid and descending colon were performed. To prevent autonomic nerve injury, TME and rectal mobilization were performed down to the pelvic floor with caution. Rectal transection was performed with a linear stapler after rectal washout. The anastomosis was completed using the double stapling technique under laparoscopic vision. Splenic flexure mobilization was carried out laparoscopically when necessary just before anastomosis. A colostomy was established under laparoscopic assistance in the Hartmann procedure.

### Definition of console time, abdominal phase, and pelvic phase

The total console time was the time from the roll-in to the roll-out of the patient cart. The abdominal phase was the period from roll-in to the completion of descending colon mobilization after IMA processing. The pelvic phase was the period from the start of rectal mobilization transection. The total console time and the duration of each phase were obtained from surgical records or videos. The time spent for lateral pelvic lymph node dissection (LLND), splenic flexure mobilization, or reinforcing sutures of the anastomosis was not included.

### CUSUM analysis

The learning curve was drawn with the CUSUM method for the total console time, the time for the abdominal phase, and the time for the pelvic phase. The CUSUM was a sequential analysis technique used to confirm slight differences in a parameter of the probability distribution [9, 10]. It detects changes over time if it is applied to the operation time. The following CUSUM score was defined in each phase.

$${\mathrm{CUSUM}}_{\mathrm n}={\mathrm X}_{\mathrm n}-{\mathrm X}_{\mathrm{mean}}+{\mathrm{CUSUM}}_{\mathrm n-1}.$$

X_n_ was the individual time, and X_mean_ was the mean of all in this formula. The CUSUM line chart was chronologically plotted. The CUSUM plots were used to determine the different stages of the learning curve. The increase in the CUSUM score showing a longer time than the average was defined as the learning phase, the staying flat showing a time almost equal to the average as the consolidation phase, and the decrease showing a shorter time than the average as the maturing phase.

The sample size was based on all available eligible cases, and a formal prospective power calculation was therefore not performed.

## Results

Table 1 shows the patient and tumor characteristics. Preoperatively, 6 (8.0%) patients underwent neoadjuvant chemoradiotherapy or chemotherapy. Further, 6 patients were in clinical stage IV (8.0%), and the median distance from the anal verge (AV) to the lower edge of the tumor was 10.0 cm (range: 5–20 cm).

**Table 1:** Patient and tumor characteristics

Variables	Values *n* = *75*
Age, median (range)	69 (43–87)
Sex, *n* (%)	
Male	43 (57.3)
Female	32 (42.7)
BMI (kg/m^2^), median (range)	22 (16–36)
ASA score, n (%)	
I	7 (9.3)
II	58 (77.3)
III	10 (13.3)
Previous abdominal surgery, *n* (%)	18 (24.0)
Neoadjuvant therapy, *n* (%)	
None	69 (92.0)
NACRT	3 (4.0)
NAC	3 (4.0)
Distance from AV (cm), median (range)	10 (5–20)
cT*, *n* (%)	
1	16 (21.3)
2	19 (25.3)
3	25 (33.3)
4	15 (20.0)
cN*, *n* (%)	
0	44 (58.7)
1	19 (25.3)
2	10 (13.3)
3	2 (2.7)
cStage*, *n* (%)	
I	32 (42.7)
II	10 (13.3)
III	27 (36.0)
IV	6 (8.0)
Adjuvant chemotherapy, *n* (%)	
Yes	16 (21.3)
No	59 (78.7)

*BMI* Body Mass Index, *ASA* American Society of Anesthesiologists, *NACRT* neoadjuvant chemoradiotherapy, *NAC* neoadjuvant chemotherapy, *AV* anal verge

*Tumors were classified according to the Japanese Classification of Colorectal, Appendiceal, and Anal Carcinoma (JCCRC)

Table 2 presents the operative and postoperative outcomes. LLND was performed in 3 (4.0%) patients. The median of the total console time was 178 (range: 60–301) min, and the total operation time was 303 (range: 187–545) min. Postoperative complications of grade ≤ II and III following the Clavien–Dindo classification were found in 20 (26.7%) and 5 (6.7%) patients, respectively (Table 3). One patient with stage IV cancer underwent surgery after liver metastasis removal.

**Table 2:** Operative and postoperative outcomes

Variables	Values *n* = *75*
Operative procedure, *n* (%)	
High anterior resection	13 (17.3)
Low anterior resection	59 (78.7)
Hartmann	3 (4.0)
Lymph node dissection*, *n* (%)	
prxD2	4 (5.3)
prxD3	71 (94.7)
LLND, *n* (%)	
Yes	3 (4.0)
No	72 (96.0)
Total operation time**, min (range)	303 (187–545)
Total console time**, min (range)	178 (60–301)
Estimated blood loss**, g (range)	0 (0–100)
Number of harvested lymph nodes** (range)	17 (3–36)
Transfusion, *n* (%)	
Yes	1 (1.3)
No	74 (98.7)
Postoperative complications, *n* (%)	
CD ≥ II	20 (26.7)
CD ≥ III	5 (6.7)
Resection status, *n* (%)	
R0	75 (100.0)
R1	0 (0.0)
Postoperative hospital stay**, days (range)	13 (9–90)

*LLND* lateral pelvic lymph node dissection, *TME* total mesorectal excision, *CD* Clavien-Dindo classification

*According to the Japanese Classification of Colorectal, Appendiceal, and Anal carcinoma

**The data are expressed as the median (range)

**Table 3:** Postoperative outcomes

	Values *n* = *75*
Postoperative complications (CD ≥ II), *n* (%)	20 (26.7)
Anastomotic leakage	6 (8.0)
Neurogenic bladder	4 (5.3)
Urinary tract infection	3 (4.0)
Paralytic ileus	2 (2.7)
Gastric ulcer	1 (1.3)
Intraabdominal abscess	1 (1.3)
Pneumonia	1 (1.3)
Small bowel obstruction	1 (1.3)
Surgical site infection	1 (1.3)
Postoperative complications (CD ≥ III), *n* (%)	5 (6.7)
Anastomotic leakage	4 (5.3)
Gastric ulcer	1 (1.3)

*CD* Clavien-Dindo classification

Table 4 shows the pathological outcomes. This study included 3 cases of neuroendocrine tumor and 1 case of papillary adenocarcinoma in addition to 71 cases of well-differentiated adenocarcinoma. The circumferential resection margin was negative (≥ 1 mm) in all cases.

**Table 4:** Pathological outcomes

	Values *n* = *75*
(y)pT*, *n* (%)	
0/is/1	22 (29.3)
2	15 (20.0)
3	34 (45.3)
4	4 (5.3)
(y)pN*, *n* (%)	
0	50 (66.7)
1	15 (20.0)
2	10 (13.3)
3	0 (0.0)
(y)pStage*, *n* (%)	
0	2 (2.7)
I	27 (36.0)
II	18 (24.0)
III	22 (29.3)
IV	6 (8.0)
Histological type, *n* (%)	
Differentiated adenocarcinoma	71 (94.7)
Undifferentiated adenocarcinoma	0 (0.0)
Others	4 (5.3)
Lymphovascular invasion, *n* (%)	
Positive	48 (64.0)
Negative	26 (34.7)
Unknown	1 (1.3)
CRM involvement, *n* (%)	
Negative	75 (100.0)
Positive	0(0.0)
DM involvement, *n* (%)	
Negative	75 (100.0)
Positive	0 (0.0)

*CRM* circumferential resection margin, *DM* distal margin

*Tumors were classified according to the Japanese Classification of Colorectal, Appendiceal, and Anal Carcinoma (JCCRC)

**Fig. 1:**
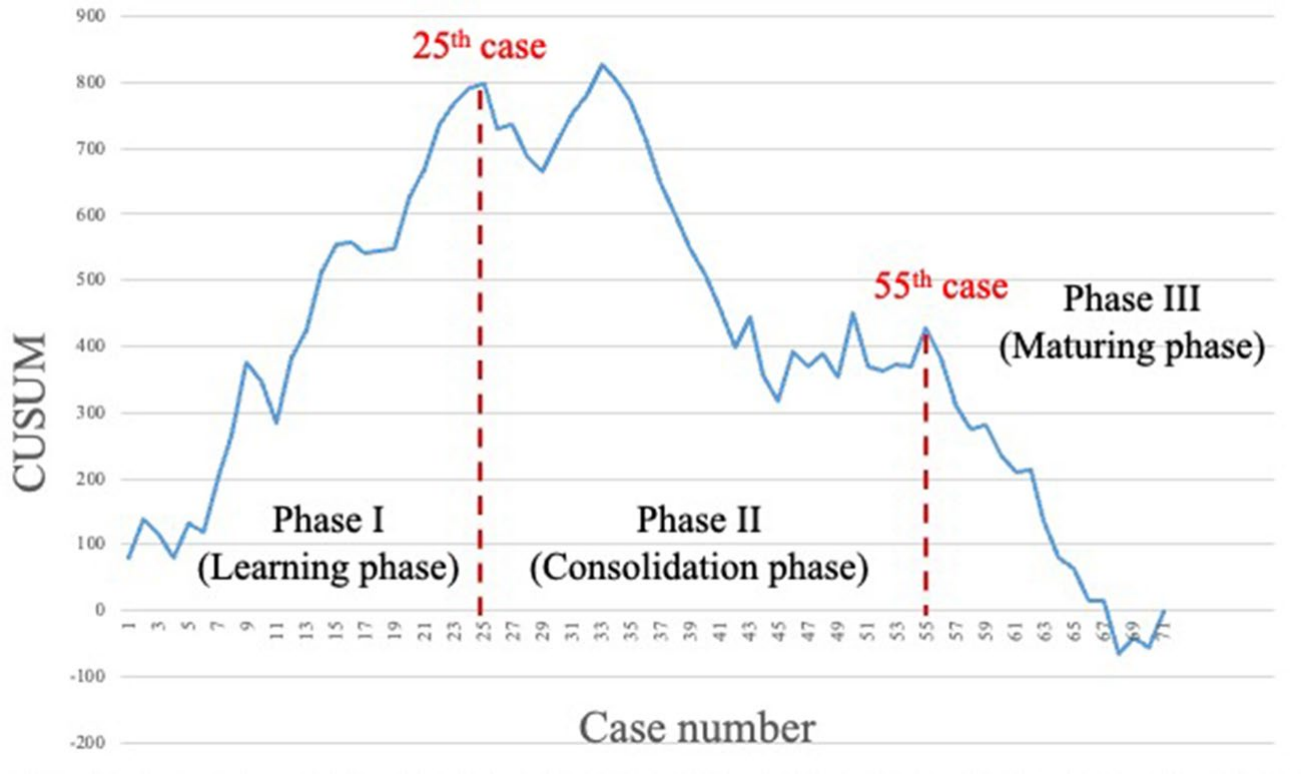
Learning curve based on the total console time. The learning curve according to the total console time consisted of three phases: I (learning phase, cases 1–25), II (consolidation phase, cases 26–55), and III (maturing phase, cases 56–71)

**Fig. 2:**
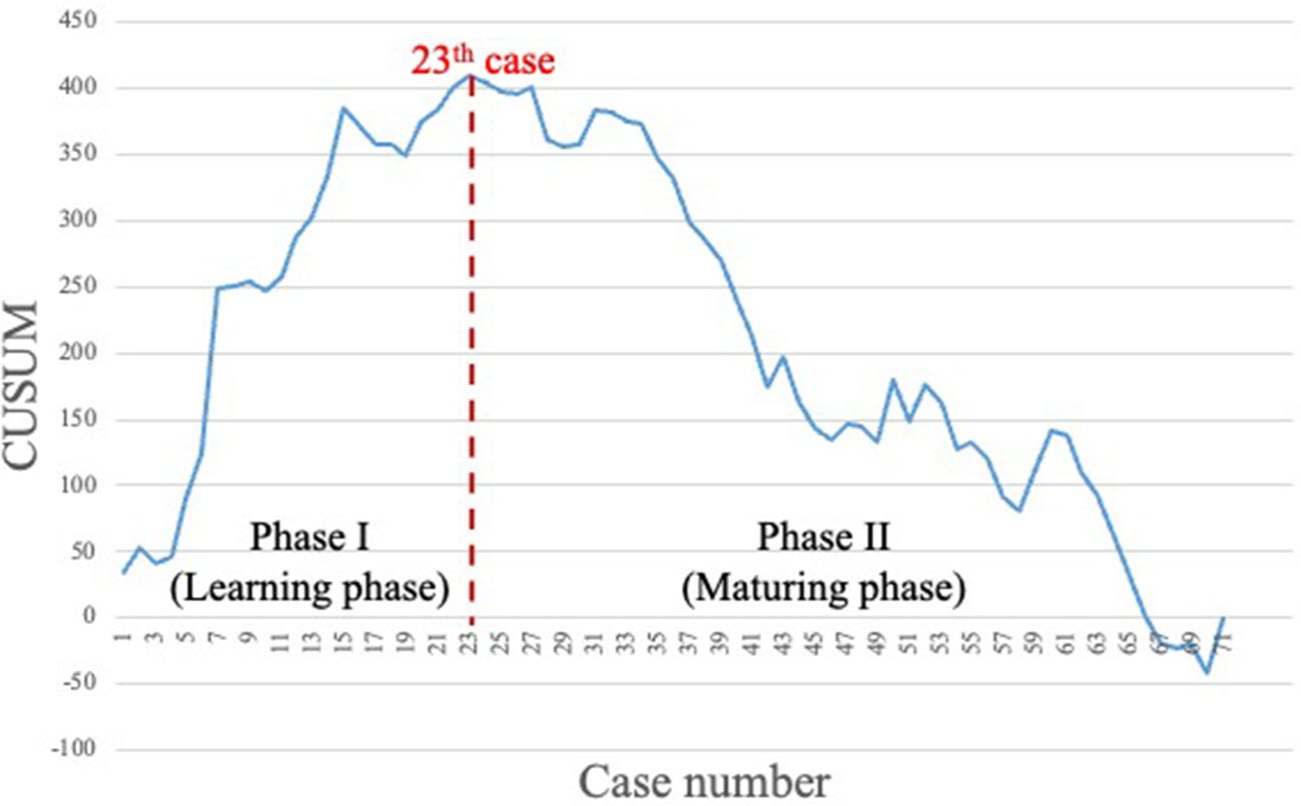
Learning curve based on the abdominal phase. In the abdominal phase, the graph was made up of two phases: I (learning phase, cases 1–23) and II (maturing phase, cases 24–71)

**Fig. 3:**
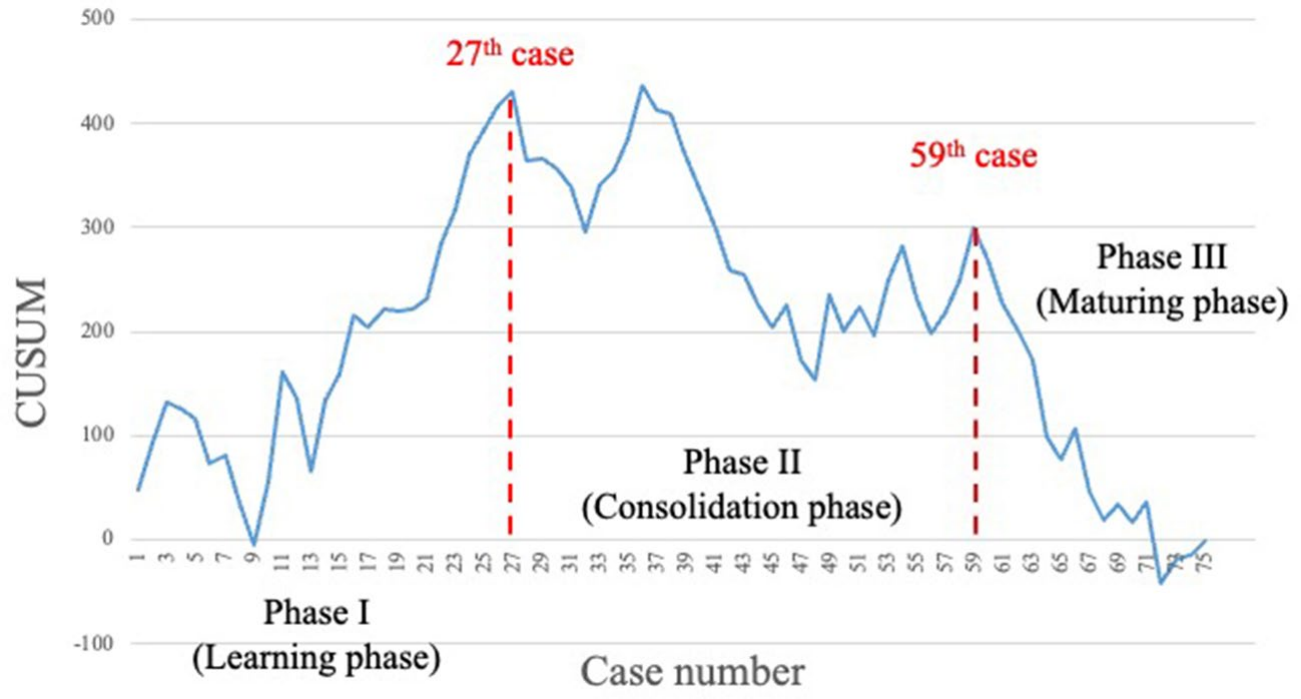
Learning curve based on the pelvic phase. In the pelvic phase, the learning curve was composed of three phases: I (learning phase, cases 1–27), II (consolidation phase, cases 28–59), and III (maturing phase, cases 60–75)

Figure 1 illustrates the learning curve for the total console time. This assessment excluded four cases from this examination in which abdominal and pelvic manipulation were performed with laparoscopy and a robot, respectively. The learning curve for the total console time consisted of three phases: I (learning phase, cases 1–25), II (consolidation phase, cases 26–55), and III (maturing phase, cases 56–71). Figure 2 shows the learning curve for the abdominal phase. The graph was made up of two phases: I (learning phase, cases 1–23) and II (maturing phase, cases 24–71) with no consolidation phase. Figure 3 illustrates the learning curve for the pelvic phase. The learning phase was achieved in 27 cases, followed by the consolidation phase in 32 cases (cases 28–59) and the maturing phase in the last 16 cases (cases 60–75). No consolidation phase was observed in the abdominal phase, whereas 32 cases were required for consolidation in the pelvic phase to reach the maturing phase.

## Discussion

RRS is becoming more popular, and junior surgeons have more opportunities to perform it [11]. Efficient education and training are required for them to learn RRS safely and quickly. The learning curve for RRS is shorter than that for laparoscopic surgery; however, the techniques required for abdominal and pelvic manipulations are different, and proficiency may vary for each [5]. In this study, we developed a learning curve of the operative time using the CUSUM method and assessed it separately for the abdominal and pelvic phases. Hence, the maturing phase of RRS was achieved after 23 and 59 cases for the abdominal and pelvic phases, respectively. No consolidation phase was found in the abdominal phase, whereas 32 cases of the consolidation phase were observed in the pelvic phase.

Pelvic manipulation requires a longer learning period than abdominal manipulation because it is affected by the tumor location, tumor size, and/or pelvic cavity width [12, 13]. The mobilization of the rectum is frequently difficult in cases with large tumor diameters. It may cause serious injuries to the autonomic nerve, ureter, prostate, and vaginal wall. Yamamoto et al. demonstrated that a tumor size of > 45 mm, an anorectal angle of > 123°, and a pelvic exit diameter of < 82.7 mm were associated with surgical difficulty during MIS [14]. Han et al. revealed the tumor distance from the AV as an independent predictor of prolonged operative time in RRS [15]. Another reason is the accumulation of effusion or blood in the pelvis, which makes anatomical recognition and surgical maneuverability challenging. Patients with advanced rectal cancer or those who have undergone neoadjuvant chemoradiotherapy often demonstrate a large amount of effusion. Suctioning must be performed through the assistant port in those cases; however, effective suctioning is sometimes difficult because the curvature of the sacrum can hinder the insertion and maneuverability of the suction tube in the pelvis [16]. The difficulty of pelvic manipulation varies by patient and tumor condition, which may take more time to mature.

The results of this study may help junior surgeons learn RRS more efficiently. Junior surgeons could benefit from a learning process that first focuses on training in abdominal manipulation, which gradually shifts to pelvic manipulation. Furthermore, training divided into phases may reduce complications and improve their technique efficiency for experienced surgeons. Sugishita et al*.* segmented the console time of RRS into three sections (time of IMA dissection, time of descending colon and sigmoid colon mobilization, and time of rectal mobilization) and developed learning curves for each of the three phases [17]. This is similar to our study, where a consolidation phase was found in the pelvic manipulation, with a different shape compared to the two learning curves for abdominal manipulation. Their study supports our results, and learning by splitting the surgical process could be effective. In contrast to the segmentation by Sugishita et al., who analyzed IMA dissection, left colon mobilization, and rectal mobilization as three separate steps, we combined IMA dissection and mobilization of the descending–sigmoid colon into a single “abdominal phase.” This is because, in our institutional workflow, these steps are performed consecutively as a continuous process without major changes in the surgical field. Moreover, dividing the procedure into only two broad phases—abdominal and pelvic—provides a simpler framework for analysis and may facilitate interpretation of the learning curve in educational settings.

In this study, the surgeries were performed with the da Vinci X or Xi Surgical System® or the hinotori™ Surgical Robot System. While the overall console handling principles are similar, surgeons might require an adjustment period for the hinotori™ system. However, Noshiro et al. reported that the console times for hinotori surgeries showed no significant learning curves with similar values to the late phase of da Vinci surgery [18]. They suggested that no additional learning curve might be required to achieve proficient surgical outcomes using the new hinotori surgical robotic platform, compared with the established da Vinci surgery.

This study has some limitations. First, this study included several surgical procedures, such as low anterior resection, high anterior resection, and Hartmann’s procedure. Therefore, we did not assess the differences in the learning curves for each surgical procedure. Another limitation is the modest case volume per surgeon at our institution. Because our tertiary center distributes cases among multiple surgeons for training purposes, the number of operations performed by each surgeon per year was relatively small. As a result, the calendar time required to progress through the learning curve phases may have been prolonged compared with higher-volume centers. Therefore, caution is needed when extrapolating the phase-specific durations observed in this study to institutions with different case volumes. Furthermore, our cohort had relatively favorable patient characteristics, with a median BMI of 22.0. This may have facilitated abdominal phase dissection by improving exposure and reducing the technical difficulty compared with patient populations in Western countries, where higher BMI is more common. Consequently, the shorter abdominal phase learning curve observed in our study may not be directly generalizable to settings with a higher prevalence of obesity. In addition, our analysis was based on predefined abdominal and pelvic phases; however, intraoperative variability exists. Anatomical variations, such as narrow pelvis, large tumors, or adhesions, may necessitate alternating between abdominal and pelvic maneuvers rather than proceeding in a strictly sequential fashion. Therefore, the phase-specific learning curves presented here may not fully capture the complexity of surgical workflow in all clinical scenarios.

In conclusion, in RRS, 23 and 59 patients were required for abdominal and pelvic manipulation, respectively, before reaching the maturing phase. The learning curve varies between the abdominal and pelvic manipulation of RRS. Building a training system that considers the different learning phases of each manipulation may enable more efficient learning of RRS.

## Data Availability

The datasets used and analyzed during the current study are available from the corresponding author on reasonable request.
